# Bone Bruises and Concomitant Meniscus and Cartilage Damage in Anterior Cruciate Ligament Injuries: A Systematic Review and Meta-Analysis

**DOI:** 10.3390/bioengineering11050515

**Published:** 2024-05-20

**Authors:** Sueen Sohn, Saad Mohammed AlShammari, Jeong Han Lee, Man Soo Kim

**Affiliations:** 1 Department of Orthopedic Surgery, Inje University Sanggye Paik Hospital, College of Medicine, Inje University, Seoul 01757, Republic of Korea; osdocsse@gmail.com; 2 King Abdulaziz Air Base Hospital, Ministry of Defense, Dhahran 34641, Saudi Arabia; s3dmohammed90@gmail.com; 3 Department of Orthopaedic Surgery, Seoul St. Mary’s Hospital, College of Medicine, The Catholic University of Korea, Seoul 06591, Republic of Korea; opnipotent@naver.com

**Keywords:** bone bruise, anterior cruciate ligament, injury, tear, meniscus, cartilage, review

## Abstract

(1) Background: Bone bruises in acute anterior cruciate ligament (ACL) injuries are closely linked to the occurrence of simultaneous meniscal and cartilage damage. Despite the frequent occurrence of associated injuries including bone bruises, meniscus, and cartilage damage in patients with ACL injuries, a systematic review of the relationships between the presence of bone bruises and the extent of meniscus and cartilage injuries has yet to be conducted. (2) Methods: Multiple comprehensive databases, including MEDLINE, EMBASE, and the Cochrane Library, were searched for studies that evaluated the relationship between bone bruises and meniscus or cartilage injuries following ACL injuries. Study selection, data extraction, and meta-analysis were performed. The Methodological Index for Non-Randomized Studies (MINORS) was used for quality assessments, and Review Manager 5.3 was used for data analysis. (3) Results: Data were extracted from 22 studies encompassing a total of 2891 patients with ACL injuries. Among the included studies, six studies investigated the relationships between bone bruises and medial meniscus (MM) or lateral meniscus (LM) injuries, while three studies investigated the relationships between bone bruises and cartilage injuries. There were no significant correlations between the presence of bone bruises and MM injuries (relative risk (RR) = 1.32; *p* = 0.61). A quantitative analysis indicated that individuals with bone bruises had a 2.71-fold higher likelihood of sustaining LM injuries than those without bone bruises (RR = 2.71; *p* = 0.0003). The analysis confirmed a significant relationship between bone bruises and cartilage injuries (RR = 6.18; *p* = 0.003). (4) Conclusions: Bone bruises occur most frequently in the lateral compartment. Bone bruises resulting from ACL injuries are related to accompanying LM injuries and cartilage injuries. Knowing these associations and the frequency of injuries may allow orthopedic surgeons to promptly address ACL-related meniscus and cartilage injuries on MRI results and in future clinical practice.

## 1. Introduction

Anterior cruciate ligament (ACL) injuries are prevalent in activities involving cutting and pivoting, with an incidence rate of 57 to 78 cases per 100,000 people each year [1,2,3,4,5,6,7,8,9,10]. Diagnosis primarily relies on physical examinations such as the pivot shift test, Lachman test, and assessments of differences in knee laxity [11]. Magnetic resonance imaging (MRI) also plays a crucial role in confirming ACL injuries and identifying associated damage like meniscal tears, articular cartilage injuries, and injuries to multiple ligaments [12,13].

Bone bruises, also known as bone marrow edema-like lesions, manifest through symptoms like hemorrhage, edema, necrosis, and fibrosis [14], and they are found in conjunction with ACL tears in approximately 80% of incidents [15]. These lesions appear as areas of heightened signal intensity in the bone marrow on T2-weighted MRI scans and are more clearly visible using techniques like fat suppression and short tau inversion recovery sequences [16]. MRI is particularly effective for detecting bone bruises, thus boasting a sensitivity of 97% for the posterior part of the lateral tibial plateau and 100% for the lateral femoral condyle [17].

Bone bruises in acute ACL injuries are closely linked to the occurrence of simultaneous meniscal and cartilage damage [18,19]. Specifically, in cases of acute ACL injury with bone bruises, 72–91% also involve meniscal injuries [18,19]. Furthermore, cartilage damage is present in 80–94% of these cases [19]. Notably, even after the resolution of bone bruises, microscopic lesions can still be detected in the adjacent joint cartilage [20].

Despite the frequent occurrence of associated injuries, including bone bruises, meniscus, and cartilage damage in patients with ACL injuries [18,19], a systematic review of the relationship between the presence of bone bruises and the extent of meniscus and cartilage injuries has yet to be conducted. In the present research, we explore potential links between bone bruises and additional meniscus and cartilage injuries in individuals suffering from ACL tears. We posited that bone bruises are indicative of concurrent meniscus and cartilage damage. Validating this hypothesis could enable orthopedic surgeons to enhance their clinical practice by incorporating these insights into diagnostic and treatment strategies.

## 2. Materials and Methods

This investigation adhered to the Preferred Reporting Items for Systematic Reviews and Meta-Analyses (PRISMA) guidelines, as outlined in the PRISMA Checklist [21]. The systematic review registration protocol was registered in a research registry. (Registration number: reviewregistry1813).

### 2.1. Data Acquisition and Literature Review

The research methodology was aligned with the principles of Cochrane Review Methods. In February 2024, extensive searches were conducted in prominent databases such as MEDLINE, EMBASE, and the Cochrane Library for English-language studies examining bone bruises post-ACL injury using detailed search terms: ‘‘(bone OR osseous) AND (bruise OR contusion OR lesion OR edema) AND (anterior cruciate ligament OR ACL).” Subsequent to the electronic search, a manual review of references and bibliographies from identified articles, including relevant reviews and meta-analyses, was undertaken to identify additional studies potentially missed in the initial search. Each article was then scrutinized for inclusion based on predefined criteria.

### 2.2. Selection of Studies

Two independent reviewers assessed the eligibility of studies based on specific inclusion criteria. Initial screening involved evaluating titles and abstracts for relevance, followed by a full-text review in cases of uncertainty. Any disagreements were resolved through discussion. Inclusion criteria specified studies including more than 15 human subjects with ACL injuries, research exploring the relationships between bone bruises and injuries to the meniscus or cartilage, use of MRI for bone bruise assessment, and documentation of bruise location in specified knee compartments—specifically the medial femoral condyle (MFC) or lateral femoral condyle (LFC) and the medial tibial plateau (MTP) or lateral tibial plateau (LTP). Only English-language articles published from 2011 to 2023 were considered. Exclusions were made for case studies, systematic reviews lacking original data, studies not specifying knee compartments for bone bruises, and cadaver studies of ACL injuries.

### 2.3. Data Extraction

Data were extracted by two reviewers using a standardized form, with any discrepancies resolved through discussion or by a third reviewer if necessary. Variables included the first author, year of publication, country, study design, MRI timing post-injury, MRI intensity, total and bone bruise-specific ACL injury sample sizes, and incidence of meniscal and cartilage injuries. Efforts were made to contact authors for additional data when necessary. A third senior investigator was consulted to resolve any disagreements during data extraction.

### 2.4. Quality Assessment

Two reviewers independently assessed the methodological quality of the included studies using the Methodological Index for Non-Randomized Studies (MINORS), with quality tiers defined by previous systematic reviews [22]. MINORS scores were independently assessed, with an ideal total score of 16 for non-comparative studies and 24 for comparative studies. The MINORS score was reported as a percentage of the ideal total score. For this review, a score of <8 was considered poor quality, 9–14 was considered moderate quality, and 15–16 was considered good quality for non-comparative studies. For comparative studies, the cutoff points were <14 for poor quality, 15–22 for moderate quality, and 23–24 for good quality [23]. Any differences in opinion regarding quality assessment were resolved through discussion among the two reviewers.

### 2.5. Statistical Analyses

Odds ratios (ORs) and 95% confidence intervals (CIs) were calculated for dichotomous outcomes. Heterogeneity was assessed with the I2 statistic, with values of 25%, 50%, and 75% considered low, moderate, and high heterogenicity, respectively. A fixed effects model was applied for I2 < 50%; otherwise, a random effects model was employed. All statistical analyses were performed using RevMan version 5.3 (The Cochrane Collaboration, Copenhagen, Denmark).

## 3. Results

### 3.1. Literature Selection

**Figure 1:**
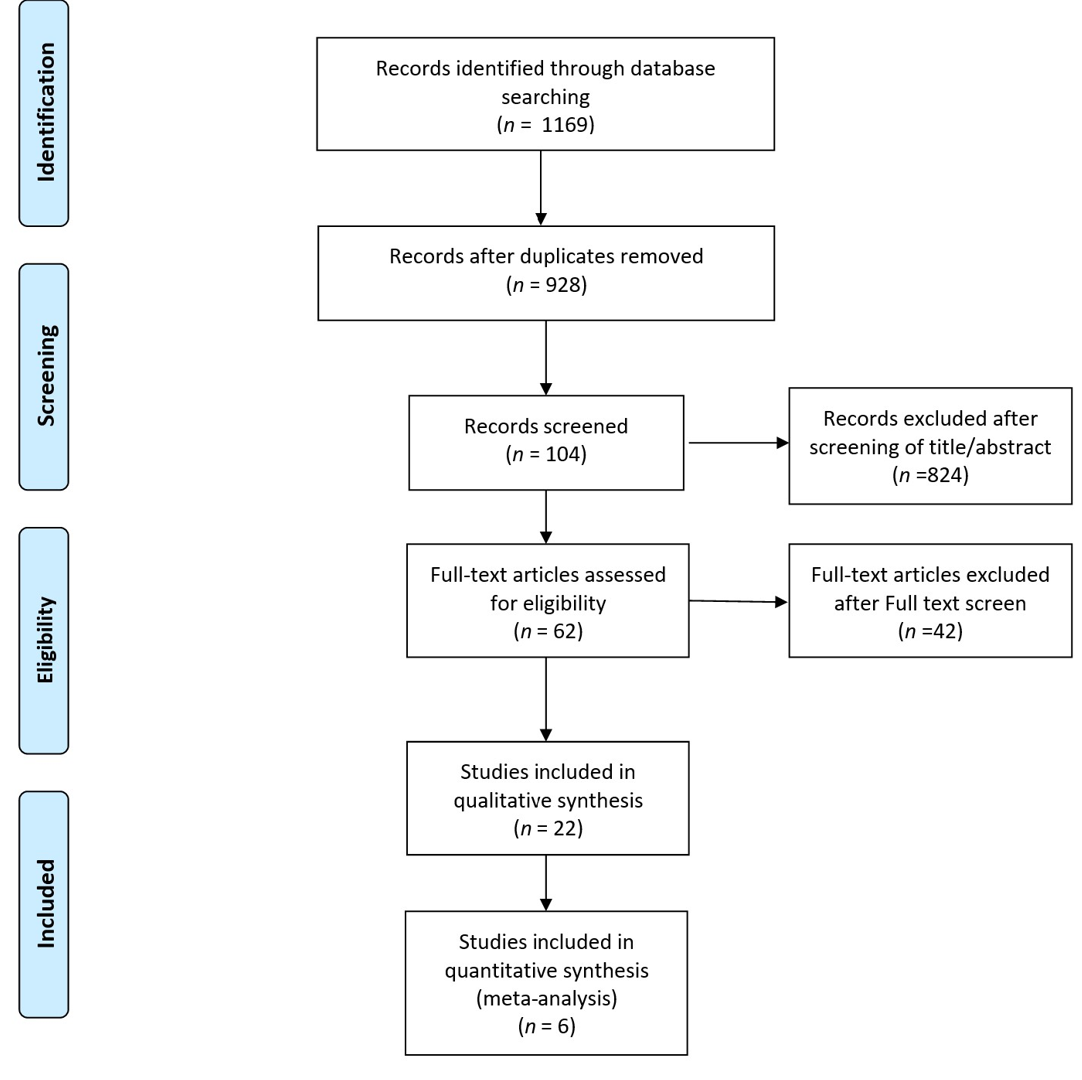
Flowchart illustrating the literature search process.

Our search strategy yielded 1169 articles, from which 241 duplicates were removed. The titles and abstracts of the remaining articles were reviewed based on predefined inclusion and exclusion criteria. This review led to the selection of 22 articles for full-text examination, thus ultimately resulting in 6 articles being included in the meta-analysis (Figure 1).

### 3.2. Study Characteristics

The data were initially extracted from 22 studies encompassing a total of 2891 patients with ACL injuries. Among the included studies, six investigated the association between bone bruises and MM or LM injuries, while three studies reported on bone bruises and cartilage injuries. All six studies were retrospective studies. The baseline characteristics of the studies are detailed in Table 1. The quality of the included studies was assessed using the MINORS scoring criteria, thus resulting in a mean score of 12.3 points in non-comparative studies and 19.9 points in comparative studies that indicated moderate study quality. These results are presented in Table 2.

### 3.3. Presence and Distribution of Bone Bruises across Anatomical Locations

Bone bruises were observed in 82.4% of all ACL injury patients (2381/2891). Upon analyzing the distribution across different anatomical sites, the lateral tibial plateau (LTP) was the most frequently affected area, with 2108 cases (88.5%), followed by the lateral femoral condyle (LFC) in 1945 cases (81.6%), the medial tibial plateau (MTP) in 1010 cases (42.4%), and the medial femoral condyle (MFC) in 847 cases (33.6%) (Table 3).

### 3.4. Characteristics of Bone Bruises and Associated Injuries

Regarding meniscus injuries, lateral meniscus (LM) injuries were most common, with a prevalence of 43.8%, which were closely followed by medial meniscus (MM) injuries at 39.6%. Cartilage injury was observed in 18.8% (181/963) of the bone bruise patients. Detailed information on the incidence of bone bruises, meniscus, and cartilage injuries is provided in Table 3.

### 3.5. Bone Bruises and MM Injuries

**Figure 2:**
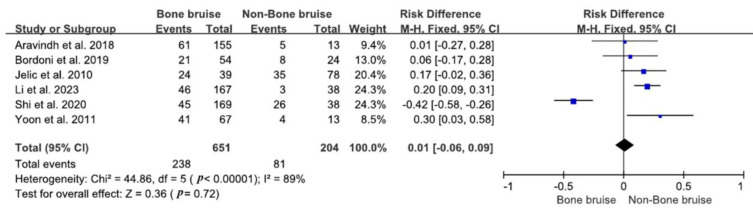
Forest plot of the associations between bone bruises and medial meniscus injuries (95% CI). The size of the blue box represents the effect size of each study included in the analysis. The diamond represents the average value of the odds ratio of studies included in the analysis, and the start and end of the diamond represent the confidence interval [18,28,35,36,38,44].

Six studies investigated the link between unspecified bone bruises and MM injuries [18,28,35,36,38,44]. In the group with bone bruises, 238 patients (36.6%, 238/651) had MM injuries compared to 81 patients (39.7%, 81/204) in the group without bone bruises. There was no significant correlation between the presence of bone bruises and MM injuries, thus indicating that individuals with bone bruises were not more likely to have MM injury than those without bone bruises (RR = 1.32; 95% CI 0.46–3.79; *p* = 0.61), as illustrated in Figure 2.

### 3.6. Bone Bruises and LM Injuries

**Figure 3:**
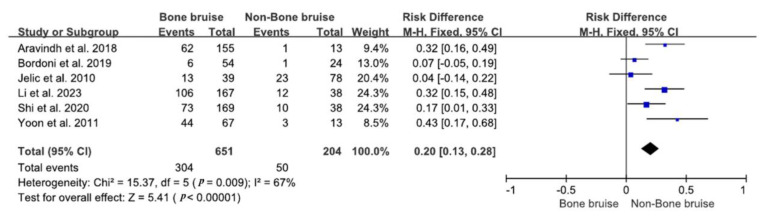
Forest plot of the associations between unclassified bone bruises and lateral meniscus injuries (95% CI). The size of the blue box represents the effect size of each study included in the analysis. The diamond represents the average value of the odds ratio of studies included in the analysis, and the start and end of the diamond represent the confidence interval [18,28,35,36,38,44].

The relationships between bone bruises and LM injuries were explored in six studies [18,28,35,36,38,44]. In these studies, LM injuries were present in 46.7% (304/651) of patients with bone bruises compared to 24.5% (50/204) in patients without such bruises. A quantitative analysis indicated that individuals with bone bruises had a 2.71-fold higher likelihood of sustaining LM injuries than those without bone bruises (RR = 2.71; 95% CI 1.58–4.67; *p* = 0.0003), as depicted in Figure 3.

### 3.7. Bone Bruises and Cartilage Injuries

**Figure 4:**
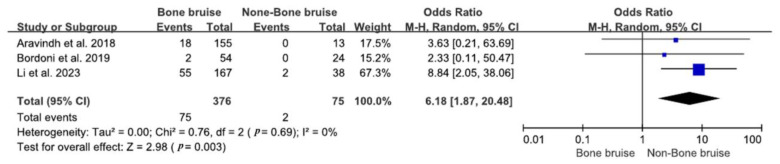
Forest plot of the associations between bone bruises and cartilage injuries (95% CI). The size of the blue box represents the effect size of each study included in the analysis. The diamond represents the average value of the odds ratio of studies included in the analysis, and the start and end of the diamond represent the confidence interval [28,36,38].

Three studies found that bone bruises in the medial or lateral compartments were associated with a higher incidence of cartilage injuries compared to individuals without bone bruises [28,36,38]. The studies explored the connection between bone bruises and cartilage injuries, thereby revealing that 19.9% (75/376) of patients with bone bruises also had cartilage injuries compared to 2.7% (2/75) in the group without bone bruises. The analysis confirmed a significant relationship between bone bruises and cartilage injuries, with individuals having bone bruises being 6.18 times more likely to have sustained cartilage injuries than those without (RR = 6.18; 95% CI 1.87–20.48; *p* = 0.003). These findings are illustrated in Figure 4.

### 3.8. The Severity of Bone Bruises and Associated Injuries

There were five studies that investigated the severity of bone bruises. Among them, three studies examined the relationship between the severity of bone bruises and concomitant injuries. Kim et al. [32] and Song et al. [40] measured the severity of bone bruise using the ICRS grade, while Bisson et al. [43] classified severity by the extent of bone bruise. Two studies found that the severity of bone bruises at the LTP was related to LM tears but not to MM tears [32,40]. However, Bissone et al. [43] showed that there was a correlation between the severity of bone bruises at the LTP and MM tears. Sone et al. [40] demonstrated that there was a correlation between the severity of bone bruises in LFC and LM tears, but there was no correlation with MM tears and cartilage injury. In a study by Kim et al. [32], it was found that there was a relationship between the severity of bone bruises at the MTP and MM tears, but there was no relationship with LM tears.

## 4. Discussion

The most important finding of this study is that the presence of bone bruises in ACL injury patients is closely related to higher incidence of LM and tibiofemoral cartilage injuries when comparing accompanying injuries in the bone bruise and non-bone bruise groups. Additionally, in patients with ACL injury, bone bruises in the medial and lateral compartments of the tibia and femur most commonly occurred in the LTP, followed by the LFC, MTP, and MFC.

For acute ACL injuries, approximately 80% of patients exhibited bone bruises on their MRI results, and the presence of BB has been linked to a higher likelihood of injuries to articular structures [18,45]. Zeiss et al. [46] reported that BB accompanied ACL injuries in up to 72% of cases, whereas only 12% of partial ACL injuries involved BB, with such cases having poorer prognosis than partial ACL injuries without BB. BB in acute ACL injuries has been attributed to bony impingement, thus suggesting that the absence of BB might indicate less knee displacement and the preservation of ACL function at the time of injury [47,48]. This implies that the force applied to the ACL might not be sufficient to cause a complete ACL tear under these conditions [46]. Conversely, the presence of BB suggests more significant knee displacement due to ACL function failure at the time of injury, thereby often resulting in a complete ACL tear and a higher chance of additional injuries [47,48]. Bone bruises following an ACL tear are predominantly found in the lateral compartment, thereby often resulting from a pivot shift valgus injury to the knee [49,50,51]. While various theories exist regarding the occurrence of bone bruises in the medial compartment, they are mainly linked to the anterior displacement of the tibia during the initial pivot shift injury [49,50,51] and the subsequent contrecoup varus force as the knee realigns post-injury [52]. These scenarios are believed to involve a significantly higher amount of energy [52]. The varus and valgus forces responsible for this coup–contrecoup injury pattern can also result in associated injuries to the ligament and meniscus [52].

In this study, the overall prevalence of bone bruises was observed to be 82.4%, which was consistent with previous studies [40,42,43,44,53,54]. The most common site of bone bruises in this study was the LTP (88.5%), followed by the LFC (81.6%), MTP (42.4%), and MFC (33.6%). Song et al. [40] analyzed bone bruises in patients with acute ACL injuries, thus finding a distribution pattern that aligns with our study. The lateral tibial plateau was the most frequently affected area (73.1%), followed by the lateral femoral condyle (60.6%). In contrast, involvement of the medial compartment was less common, with the MTP affected in 21.2% of cases and the MFC in only 6.2% of cases. This is attributed to a typical injury mechanism involving anterior displacement of the LTP relative to the LFC and the application of valgus stress [49,51].

ACL injuries frequently occur alongside other intra-articular structural damage [42,54]. Recognizing associated injuries is crucial for thorough evaluation during MRI and arthroscopic surgery [42,54]. The reported incidences of MM and LM injuries in the acute phase following an ACL injury range from 15% to 73% and 10% to 55%, respectively [55,56,57,58]. Illingworth et al. [53] found that between 65% and 70% of adults with a bone bruise following an ACL tear also had a meniscal tear. In our study, 39.6% of patients exhibited MM injuries, and 43.8% had LM injuries, thus aligning with the findings of previous research [40,44,59]. Chondral injuries, which have been reported in up to 15% of patients in other studies, were identified in 18.8% in this review, thus indicating a slightly higher prevalence in the meta-analysis patient cohort.

Our findings indicate a significant correlation between the presence of bone bruises and LM injuries, thereby echoing the results of prior research [43,44]. Spindler et al. [59] studied 44 patients with acute ACL injuries and found that 68% exhibited bone contusion on the LFC. Their arthroscopic evaluations revealed that 56% of these patients also had LM lesions. This finding aligns with those of Yoon et al. [44], who observed an increasing trend in the prevalence of LM lesions with more extensive lateral bone contusions on MRI results in patients with ACL injuries. The characteristic pattern of bone bruises in the lateral compartment suggests that the LM is trapped and compressed between the femur and tibia during injury, thus likely contributing to the occurrence of LM injuries [43,44]. There are various results regarding the relationship between bone bruises and meniscus injury in ACL injury patients [43,44,60,61]. Our results confirmed the relationship between bone bruises and LM injury, but they did not confirm a relationship between bone bruises and MM injury. Bisson et al. demonstrated that lateral bone bruises were linked to LM tears, and more severe LTP bruising was correlated with MM tears [43]. Calvo et al. found associations between bone bruising on the MTP and tears in the posterior medial meniscus [61]. Bastos et al. [60] argued that bone bruising should not be seen as indicative of meniscus tear severity or used to infer injury severity, thus suggesting it might be overemphasized in assessments. Therefore, the relationship between bone bruises and meniscus injuries remains controversial, and it seems necessary to comprehensively review more research results [43,44,60,61].

Prior studies have shown that in adults with ACL tears, the presence of a bone bruise is often associated with damage to the corresponding cartilage layer, with 59% to 80% of such adults also exhibiting cartilage injuries [62,63]. In the present study, we also found that bone bruising and cartilage injury are closely related. In ACL injuries accompanied by bone bruises, the possibility of cartilage damage due to bone contusion due to much greater trauma increases compared to ACL injuries without bone bruises [49,54]. This relationship may be different in pediatric patients [36]. We included three studies in our meta-analysis to investigate the relationship between bone bruising and cartilage injury in ACL injury patients [28,36,38]. Among these, one study was conducted solely among pediatric patients [36]. In the other two studies, involving both adult and pediatric patients, the incidence of cartilage injury was 10.7–32.9% among bone bruise patients [28,38]. However, this correlation does not hold in the pediatric population [36], as observed in the current study. Among the young patients with bone bruises, only 3.7% also had cartilage injuries [36]. The lower prevalence of cartilage damage in children and adolescents may be due to lower-energy traumas compared to adults [64]. Additionally, the joint tissues of younger individuals are more elastic and resilient to traumatic injuries [65,66]. Pediatric cartilage, which varies in thickness based on factors such as sex, weight, and physical activity, is generally thicker than that of adults, thus potentially offering greater protection against injury [65,66]. Therefore, it will be necessary to clearly investigate the relationship between bone bruising and cartilage injury by distinguishing between children and adults in future studies [28,36,38].

Several limitations of the present study should be mentioned. First, in systematic reviews, the quality of the original data can limit the overall quality of the research. All studies were retrospective, thus indicating a need for more prospective research in this area. Second, the inclusion of only published data might introduce reporting bias, given the propensity for negative outcomes to be less frequently reported. Third, while it is crucial to compare patients with and without bone bruises to elucidate the relationship between bone bruises and injuries to the meniscus and cartilage, it is also vital to further investigate the relationship between the specific locations of bone bruises and associated injuries. However, in this study, we were unable to examine the relationships between the specific locations of bone bruises and damage to intra-articular structures. Fourth, we lacked data to confirm the relationship between bone bruises and cartilage damage in this study, which may limit the accuracy of our results. Future research will likely be required to synthesize additional findings and clearly present outcomes. Fifth, a limitation of research related to bone bruises in ACL injuries is the inability to control the timing from injury to MRI imaging. The timing of MRI examinations is a major factor affecting the reliability of bone contusion patterns. A recent study indicated that the incidence of bone contusions was over 80% during the acute phase (within 6 weeks of injury) and the subacute phase (6 weeks to 3 months after injury), but this rate decreased by 57% in the intermediate phase (between 3 months and 1 year after injury) [67]. Therefore, in this study, the time from injury to MRI was set at 3 months, which is considered the upper limit of the “acute” injury standard [33,67]. Most of the studies included in this systematic review had MRI scans performed within 6 weeks of the ACL injury, and there were two studies where scans were taken at 3 months [33,67]. Sixth, the association between concomitant injuries and bone bruises by subdividing the locations of the bone bruises was not investigated in this study. Generally, the compartment distribution of bone bruises is also related to the occurrence of concomitant injuries in ACL injury [38]. It is known that bone bruises in the lateral compartment are associated with LM injuries [38]. The pattern of associated injuries may vary depending on the location of the bone bruise in ACL injury. Further research is needed in this regard. Finally, no distinction was made regarding gender [43], age [29,31], or types of injury, thus including both contact and non-contact injuries [32]. Although these factors can significantly influence the pattern of bone bruising, they could not be distinguished using the data included in this study; hence, they were aggregated.

Various studies have been conducted on bone bruises in ACL injury [8,24,25,26,29,30,33,43,68,69,70]. In addition to research on bone bruises and associated injury in ACL injury [69,70], there were also studies on the ACL injury mechanism according to the bone bruise pattern [68]. However, most studies focused on the relationship between ACL bone bruises and ligament injuries [71], and there were no systematic reviews or meta-analyses that synthesized the findings from several studies on the relationship between ACL bone bruises and meniscus or cartilage injuries. Previously, there were individual studies that reviewed only the bone bruise patterns and resultant injuries [8,24,25,26,29,30,33,43,68,69,70], but this research compiled numerous studies on the relationships among bone bruises, meniscal injuries, and cartilage damages. By investigating the relationships between bone bruises, as well as meniscal and cartilage damages in ACL injuries, our findings provide additional explanations to patients about the associated injuries in clinical settings, thus enhancing clinical relevance.

## 5. Conclusions

Bone bruises occur most frequently in the lateral compartment. Bone bruises resulting from ACL injuries are related to accompanying LM injuries and cartilage injuries. However, to confirm this more clearly, high-quality, large-scale cohort studies are needed to examine the association with concomitant injuries by subdividing the bone bruise patterns in ACL injuries. Knowing these associations and the frequencies of injuries may allow orthopedic surgeons to promptly address ACL-related meniscus and cartilage injuries on MRI results and in future clinical practice.

## Figures and Tables

**Table 1:** Characteristics of included studies.

Author	Year	Nation	Period	Study Design	MRI Timing	MRI Intensity	Sample Size	Bone Bruise	Age, Years	Gender (M)
Wang et al. [24]	2023	China	2011–2020	Retrospective	4 wks	1.5 T	188	153	15.2	55
Vermeijden et al. [25]	2023	Netherland	2019	Retrospective	31 days	1.5 or 3 T	254	251	34	114
Orringer et al. [26]	2023	USA	2015–2021	Retrospective	8 wks		26	20	11.8	17
							26	20	34.3	17
Moran et al. [27]	2023	USA		Retrospective	30 days	3 T	78	75	23.1	54
							142	138	25.6	77
Li et al. [28]	2023	China	2021–2022	Retrospective	3 wks	1.5 T	205	167	27.05	118
Galloway et al. [29]	2023	USA	2014–2019	Retrospective	2 months		60	57	23.4	31
D’Hooghe et al. [30]	2023	Italy	2014–2018	Retrospective			19	19	19.5	19
Brophy et al. [31]	2023	USA	2015–2019	Retrospective	3 weeks		191	181		
Kim et al. [32]	2022	Japan	2013–2021	Retrospective		2 T	176	141	26.8	98
Agostinone et al. [33]	2022	Italy		Retrospective	3 months	1.5 T	29	24	29.1	24
Shi et al. [34]	2021	China	2016–2018	Retrospective	4 wks	1.5 T	56	43	30.3	2
							82	66	26.7	6
Shi et al. [35]	2020	China	2016–2018	Retrospective	4 wks	1.5 T	207	169	28.7	
Bordoni et al. [36]	2019	Swiss	2010–2018	Retrospective	90 days		78	54	14.3	41
Novaretti et al. [37]	2018	USA	2012–2016	Retrospective	6 wks		53	51	13.3	26
Aravindh et al. [38]	2018	Singapore	2013–2016	Retrospective	6 wks		168	155		126
Lattermann et al. [39]	2017	USA		Retrospective			81	81		
Song et al. [40]	2016	China	2011–2013	Retrospective	6 wks	1.5 T	193		32.3	141
Filardo et al. [41]	2015	Italy	2004–2008	Retrospective	1 month		134	74	31.9	98
Witstein et al. [42]	2014	USA	2005–2010	Retrospective	6 wks	1.5 T	73	70		28
Bisson et al. [43]	2013	USA	2005–2011	Retrospective	6 wks	1.5 T	171	154	25.2	89
Yoon et al. [44]	2011	Korea	2006–2008	Retrospective	6 wks		81	68	29	22
Jelic et al. [18]	2010	Serbia		Retrospective	1 month	0.3 T	120	39	31	88

**Table 2:** Quality assessment of the included studies.

Author	Clearly Stated Aim	Inclusion of Consecutive Patients	Prospective Collection of Data	Endpoints Appropriate for Aim	Unbiased Assessment of Endpoints	Appropriate Follow-Up Period	Lost to Follow-Up< 5%	Prospective Calculation of Study Size	Item 9–12 Only for Comparative Studies	Adequate Control Group	Contemporary Groups	Baseline Equivalence of Groups	Adequate Statistical Analysis	Total Score
Wang et al. [24]	2	2	0	2	2	2	2	0						12
Vermeijden et al. [25]	2	2	0	2	2	2	2	1		2	2	2	2	21
Orringer et al. [26]	2	2	0	2	2	2	2	0		2	2	2	2	20
Moran et al. [27]	2	2	0	2	2	2	2	0		2	2	2	2	20
Li et al. [28]	2	2	0	2	2	2	2	1		2	2	2	2	21
Galloway et al. [29]	2	2	0	2	2	2	2	1						13
D’Hooghe et al. [30]	2	2	0	2	2	2	2	0						12
Brophy et al. [31]	2	2	0	2	2	2	2	0		2	2	2	2	20
Kim et al. [32]	2	2	0	2	2	2	2	1		1	1	2	2	19
Agostinone et al. [33]	2	2	0	2	2	2	2	1		2	2	2	2	21
Shi et al. [34]	2	2	0	2	2	2	2	0		2	2	2	2	20
Shi et al. [35]	2	2	0	2	2	2	2	0		2	2	2	2	20
Bordoni et al. [36]	2	2	0	2	2	2	2	0						12
Novaretti et al. [37]	2	2	0	2	2	2	2	0		2	2	2	2	20
Aravindh et al. [38]	2	2	0	2	2	2	2	0		2	2	2	2	20
Lattermann et al. [39]	2	2	0	2	2	2	2	0		2	2	2	2	20
Song et al. [40]	2	2	0	2	2	2	2	1		2	2	2	2	21
Filardo et al. [41]	2	2	0	2	2	2	2	0		2	2	2	2	20
Witstein et al. [42]	2	2	0	2	2	2	2	0		2	2	2	2	20
Bisson et al. [43]	2	2	0	2	2	2	2	0		2	2	2	2	20
Yoon et al. [44]	2	2	0	2	2	2	2	0		1	1	2	2	18
Jelic et al. [18]	2	1	0	1	2	2	2	0		2	1	2	2	17

**Table 3:** Bone bruise prevalence, medial and lateral meniscus, and cartilage injury prevalence.

Author	ACL Sample	Bone Bruise Sample	LTP	MTP	LFC	MFC	MM	LM	Cartilage
Wang et al. [24]	188	153	139	48	136	40	59	58	22
Vermeijden et al. [25]	254	251	240	32	163	138	79	72	
Orringer et al. [26]	26	20	18	1	19	4	11	10	
	26	20	16	9	10	12	16	11	
Moran et al. [27]	78	75	70	47	65	49	28	27	
	142	138	77	102	119	120	31	59	
Li et al. [28]	205	137	167	90	135	62	46	106	55
Galloway et al. [29]	60	57	53	16	46	13	28	30	8
D’Hooghe et al. [30]	19	19	18	3	12	0	9	6	2
Brophy et al. [31]	191	181	154	93	140	44	78	113	
Kim et al. [32]	176	141	82	47	116	29	56	42	7
Agostinone et al. [33]	29	24	24	16	21	5	12	8	4
Shi et al. [34]	56	43	40	32	38	12	12	17	
	82	66	62	31	42	20	23	27	
Shi et al. [35]	207	169	169	80	156	91	45	73	
Bordoni et al. [36]	78	54	44	11	57	34	21	6	2
Novaretti et al. [37]	53	51	51	37	51	20	10	19	
Aravindh et al. [38]	168	155	141	95	132	50	61	62	18
Lattermann et al. [39]	81	81	76	46	66	20	42	42	17
Song et al. [40]	193	141	141	41	117	12	84	94	20
Filardo et al. [41]	134	74	35	11	23	5	34	11	
Witstein et al. [42]	73	70	67	45	70	31	34	29	
Bisson et al. [43]	171	154	145	44	132	11	59	65	26
Yoon et al. [44]	81	68	59	21	55	19	41	44	
Jelic et al. [18]	120	39	20	12	24	6	24	13	
Total	2891	2381	2108	1010	1945	847	943	1044	181

## Data Availability

The data presented in this study are available in the main article.
